# Transmembrane Protein PTCHD4 Is a Novel Regulator of Cellular Senescence and Age‐Related Pathologies

**DOI:** 10.1111/acel.70711

**Published:** 2026-09-11

**Authors:** Mingyue Wang, Zhaoyun Yang, Qikai Wang, Zeping Li, Zhihua Huang, Le Wang, Xiaoning Sun, Donglin Yu, Wenhua Yu, Xiaoxia Bao, Zebin Mao, Yang Li

**Affiliations:** ^1^ Department of Biochemistry and Molecular Biology, School of Basic Medical Sciences Peking University Beijing China; ^2^ Sino‐Danish College, Sino‐Danish Center for Education and Research University of Chinese Academy of Sciences Beijing China; ^3^ Key Laboratory of Gastrointestinal Cancer, Ministry of Education, School of Basic Medical Sciences Fujian Medical University Fuzhou Fujian Province China; ^4^ Department of Clinical Laboratory Peking University First Hospital Beijing China; ^5^ Affiliated Hospital of Liaoning University of Traditional Chinese Medicine Shenyang Liaoning Province China; ^6^ Department of Gynecology, Dongzhimen Hospital Beijing University of Chinese Medicine Beijing China; ^7^ Department of Cell Biology, School of Basic Medical Sciences Peking University Beijing China

## Abstract

Cellular senescence is a key driver of age‐related pathologies. Our study focuses on PTCHD4, a transmembrane protein with previously undefined physiological functions in aging processes. We found that PTCHD4 expression was increased across multiple cellular senescence models and showed an age‐associated increase in mouse tissues and human lung transcriptomic datasets. Functionally, PTCHD4 deficiency attenuated senescence progression, whereas its overexpression promoted this process. In vivo, PTCHD4 deficiency alleviated D‐galactose‐induced aging‐related phenotypes and functional deterioration, accompanied by an extension of median lifespan in mice. In a bleomycin‐induced pulmonary fibrosis model, PTCHD4 deficiency reduced collagen deposition, attenuated senescence‐ and inflammation‐associated signals, and preserved pulmonary function. Mechanistically, PTCHD4 promoted aging‐associated AKT activation, and restoration of AKT signaling reversed the anti‐senescent effects of PTCHD4 deficiency. Taken together, these findings support PTCHD4 as a contributor to cellular senescence and age‐associated tissue dysfunction, and suggest that the PTCHD4‐AKT axis warrants further investigation as a candidate target in senescence‐associated diseases.

## Graphical abstract

PTCHD4 is induced by aging‐ and injury‐associated stresses and acts as a senescence‐associated transmembrane regulator. Through AKT activation, PTCHD4 promotes cellular senescence, SASP, functional decline, and pulmonary fibrosis, whereas PTCHD4 deficiency dampens AKT signaling and alleviates senescence‐associated pathological phenotypes.

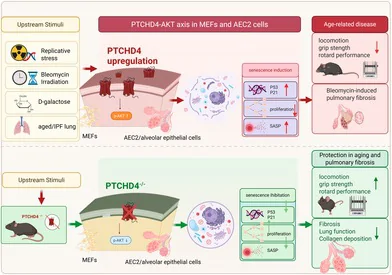

## 1 Introduction

Cellular senescence is a stress‐induced state of stable proliferative arrest characterized by increased senescence‐associated β‐galactosidase (SA‐β gal) activity, impaired DNA synthesis, and induction of p21, p53, and p16^INK4A^ (Ogrodnik 2021). The age‐dependent accumulation of senescent cells drives tissue dysfunction primarily through the senescence‐associated secretory phenotype (SASP), which promotes chronic inflammation and contributes to diverse age‐related diseases, including neurodegeneration, osteoporosis, atherosclerosis, osteoarthritis, and fibrosis (de Magalhães 2024; Bussian et al. 2018; Farr et al. 2017; Childs et al. 2016; Jeon et al. 2022; Schafer et al. 2017). Current therapeutic strategies primarily rely on both synthetic and naturally occurring senolytics, including BCL‐2 family inhibitors (Yosef et al. 2016; Zhu et al. 2016; He et al. 2020), mechanistically distinct agents and naturally occurring flavonoids, have been shown to alleviate senescence‐associated tissue dysfunction. However, their limited specificity and intrinsic toxicity constrain their long‐term and safe clinical application. In addition to senolytic molecules, immunotherapeutic strategies have emerged as alternative approaches for treating age‐related diseases, including CAR‐T cells (Amor et al. 2020; Yang et al. 2023), NK cell (Ming et al. 2025; Bai et al. 2022; Basar et al. 2020; Cheng et al. 2023), B cell (Schloss et al. 2022) based therapies and vaccines (Wu et al. 2024; Dugan et al. 2020; Yoshida et al. 2020; Yu et al. 2025; Suda et al. 2021) that primarily target surface molecules highly expressed on senescent cells to enable the selective elimination of senescent cells. These advances highlight the growing need for more precise and targeted interventions in age‐related diseases driven by cellular senescence.

PTCHD4 is a transmembrane protein belonging to the patched domain family, structurally related to PTCH1, a well‐established inhibitor of Hedgehog signaling (Huang et al. 2019; Harvey et al. 2014). However, its physiological function of PTCHD4 remains largely undefined (Schloss et al. 2022; Wu et al. 2024). In this study, we identify PTCHD4 as a previously unrecognized regulator of cellular senescence. PTCHD4 is upregulated in senescent cells and contributes to the development of senescence‐associated phenotypes in cellular models, with supportive evidence from mouse models. These findings suggest that PTCHD4 is a candidate senescence‐associated molecule with potential relevance for future mechanistic and translational studies.

## 2 Results

### 2.1 PTCHD4 Is Upregulated During Cellular Senescence and Tissue Aging

**FIGURE 1:**
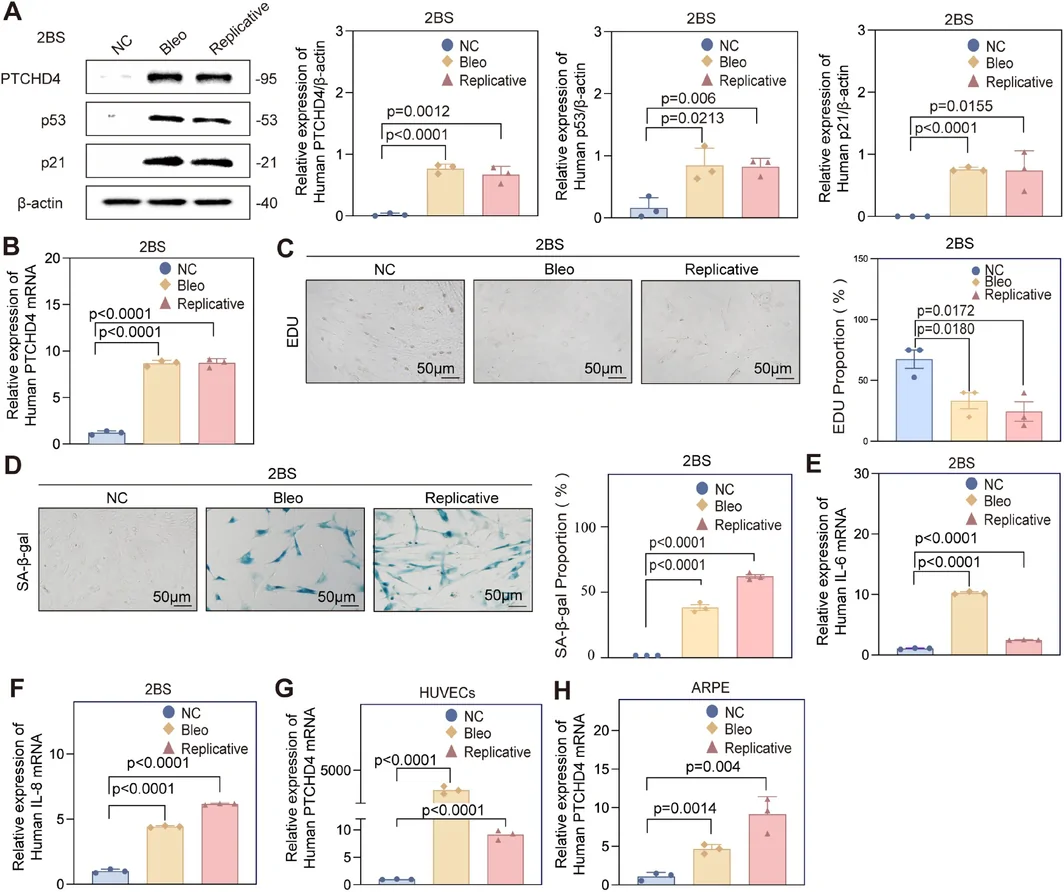
PTCHD4 expression increases during cellular senescence. (A) Representative Western blot images and quantification of PTCHD4, p53, and p21 protein levels in 2BS cells treated under NC, Bleo‐induced senescence (Bleo), or replicative senescence (Replicative) conditions (*n* = 3). (B) RT‐qPCR analysis of PTCHD4 mRNA expression in 2BS cells treated as indicated (NC, Bleo, and Replicative, *n* = 3). (C) Representative images and quantification of EdU staining in 2BS cells treated as indicated (NC, Bleo, and Replicative, *n* = 3). (D) Representative images and quantification of SA‐β gal staining in 2BS cells treated as indicated (NC, Bleo, and Replicative, *n* = 3). (E, F) RT‐qPCR analysis of IL‐6 and IL‐8 mRNA levels in 2BS cells treated as indicated (NC, Bleo, and Replicative, *n* = 3). (G, H) RT‐qPCR analysis of PTCHD4 mRNA expression in HUVEC and ARPE cells treated as indicated (NC, Bleo, and Replicative, *n* = 3).

To investigate the expression of PTCHD4 during cellular senescence, we first analyzed its expression across multiple senescence models. In human fetal lung diploid fibroblasts (2BS), PTCHD4 mRNA and protein levels were significantly increased under genotoxic stress (bleomycin treatment) and replication stress conditions, compared to young proliferating controls (Figure 1A,B). This upregulation was accompanied by the manifestation of senescence‐associated phenotypes, including reduced EdU incorporation, increased SA‐β gal activity (Figure 1C,D), elevated expression of senescence markers (p53 and p21) (Figure 1A), and upregulation of SASP factors such as IL‐6 and IL‐8 (Figure 1E,F). Similar increases in PTCHD4 expression were observed in mouse embryonic fibroblasts (MEFs) during senescence, suggesting that elevation of PTCHD4 is not species‐specific (Figure S1A–F).

Given the important contribution of alveolar epithelial type II cells (AEC2s) to pulmonary fibrosis and epithelial cell senescence, we further examined PTCHD4 expression in AEC2s after senescence induction. Both irradiation and bleomycin treatment markedly increased PTCHD4 protein and mRNA levels in AEC2s, accompanied by elevated expression of p53 and p21, decreased EdU incorporation, increased SA‐β‐gal positivity, and enhanced expression of SASP factors IL‐6 and IL‐8 (Figure S2A–F). These results indicate that PTCHD4 is also upregulated during AEC2 senescence and may be associated with epithelial senescence‐related pathological processes. Furthermore, human umbilical vein endothelial cells (HUVECs) and adult retinal pigment epithelial cells (ARPE‐19) exhibited significant PTCHD4 mRNA upregulation following senescence induction by genotoxic stress (bleomycin treatment) and replication stress (Figure 1G,H), demonstrating that PTCHD4 upregulation is a universal feature of senescent cells that is cell‐type independent.

Transcriptome data from the Aging Atlas (https://ngdc.cncb.ac.cn/aging/index) further confirmed the upregulation of PTCHD4 in IR‐induced and replicative senescent human diploid fibroblasts (WI38, IMR90) and human arterial endothelial cells (HAECs) (Figure S3A–C). Importantly, PTCHD4, a disease‐associated molecule, is typically expressed at low or undetectable levels in normal cells and tissues under physiological conditions, as documented in the NCBI database (GSE64283). This observation led us to hypothesize that PTCHD4 might potentially have a substantial impact on cellular senescence.

**FIGURE 2:**
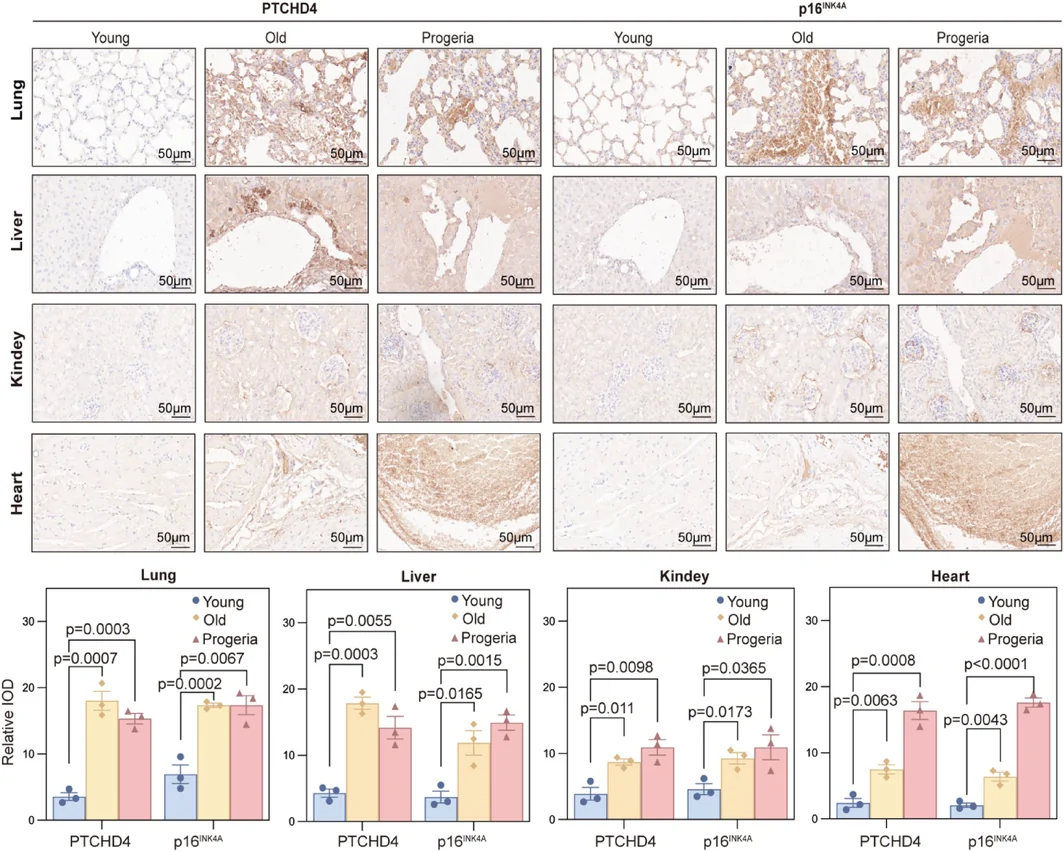
PTCHD4 expression increases in tissues of aged mice. Representative IHC images and quantification of PTCHD4/p16^INK4A^‐positive areas in the lung, liver, kidney, and heart of young WT (8‐week‐old), old WT (90‐week‐old), and progeria (12‐week‐old) mice (*n* = 3). Scale bars, 50 μm.

To examine the tissue expression of PTCHD4 in aged mice, lung, liver, kidney, and heart tissues were collected from young wild‐type (WT) mice (8 weeks), old WT mice (90 weeks), and progeroid mice (Zmpste24^−/−^, 12 weeks). Immunohistochemical (IHC) staining was performed to assess PTCHD4 expression, with p16^INK4A^ serving as a marker of cellular senescence for comparison. PTCHD4 staining was increased in the examined lung, liver, kidney, and heart tissues from old WT and progeroid mice, showing a pattern broadly consistent with p16^INK4A^ staining (Figure 2). These findings suggest an association between PTCHD4 expression and tissue aging in vivo.

To further evaluate the relevance of PTCHD4 to human lung aging and fibrosis, we analyzed multiple public human lung datasets. The proportion of PTCHD4‐positive AT2/ATII cells was increased in IPF samples compared with controls across independent datasets (Figure S4A–C). In the GSE136831 dataset, aberrant basaloid cells were enriched in IPF lungs among AT2 and aberrant basaloid epithelial cells (Figure S4D). Moreover, PTCHD4 expression or the proportion of PTCHD4‐positive AT2 cells showed an age‐associated increase in human lung datasets (Figure S4E–H). Notably, PTCHD4‐positive epithelial cells, including aberrant basaloid cells in IPF lungs, exhibited higher expression of senescence‐related markers and increased SASP module scores compared with PTCHD4‐negative cells (Figure S4I–K). These findings further support an association between PTCHD4 expression, epithelial senescence, aging, and IPF‐related pathological remodeling in human lungs.

### 2.2 PTCHD4 Regulates the Progression of Cellular Senescence

**FIGURE 3:**
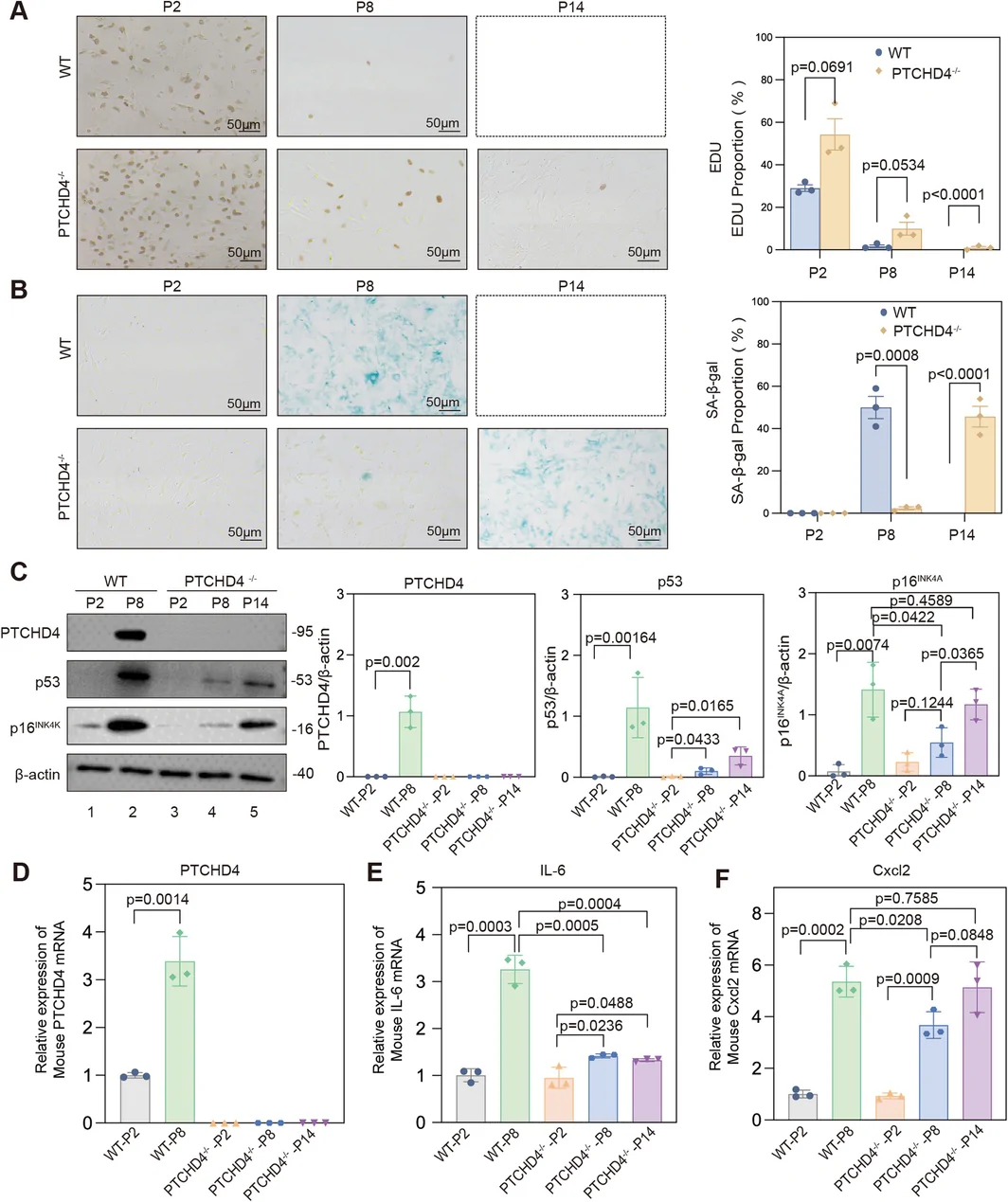
PTCHD4 deficiency delays cellular senescence. (A) Representative images and quantification of EdU staining in WT and PTCHD4^−/−^ MEFs at the indicated passages (P2, P8, P14, *n* = 3). (B) Representative images and quantification of SA‐β gal staining in WT and PTCHD4^−/−^ MEFs at P2, P8 and P14 (*n* = 3). (C) Representative western blot images and quantification of PTCHD4, p53 and p16^INK4A^ protein levels in WT and PTCHD4^−/−^ MEFs at P2, P8 and P14 (*n* = 3). (D) RT‐qPCR analysis of PTCHD4 mRNA expression in WT and PTCHD4^−/−^ MEFs at P2, P8 and P14 (*n* = 3). (E, F) RT‐qPCR analysis of IL‐6 and Cxcl2 mRNA expression in WT and PTCHD4^−/−^ MEFs at P2, P8 and P14 (*n* = 3).

To investigate the role of PTCHD4 in cellular senescence, we isolated MEFs from PTCHD4−/− mice, using MEFs from WT mice as controls. To assess the impact of PTCHD4 deficiency on cellular responses to replicative stress, we induced senescence by continuous passaging. The results showed that WT MEFs exhibited marked growth arrest and extensive senescence at passage 8, as evidenced by a significant reduction in EdU incorporation (Figure 3A) and pronounced SA‐β gal positivity (Figure 3B), and increased IL‐6, p53, and p16^INK4A^ expression (Figure 3C–G). Notably, PTCHD4^−/−^ MEFs maintained their proliferative capacity until passage 14, displaying senescent characteristics comparable to those of WT MEFs at passage 8. Taken together, these findings suggest that PTCHD4 deficiency can effectively mitigate senescence.

To complement these loss‐of‐function experiments, we overexpressed PTCHD4 in young WT MEFs. Western blot analysis showed that PTCHD4 expression was significantly upregulated in PTCHD4‐overexpressing (PTCHD4‐OE) MEFs compared with young cells, indicating successful overexpression (Figure S5A). The results showed that PTCHD4‐OE MEFs exhibited markedly reduced EdU incorporation (Figure S5B) and increased SA‐β gal activity (Figure S5C) by early passage 5. Besides, at this passage, the expressions of senescence‐associated markers p53, p16, and IL‐6 were also significantly elevated (Figure S5D–G); this suggests that PTCHD4 plays a crucial regulatory role in the progression of cellular senescence.

Given that PTCHD4 was also upregulated during AEC2 senescence, we further examined whether PTCHD4 overexpression could directly induce senescence‐associated phenotypes in AEC2 cells. PTCHD4 overexpression markedly increased PTCHD4 protein and mRNA levels in AEC2 cells, accompanied by elevated expression of the senescence markers p53 and p21 (Figure S6A,B). Functionally, PTCHD4‐overexpressing AEC2 cells showed significantly decreased EdU incorporation and increased SA‐β‐gal positivity, indicating reduced proliferative capacity and enhanced cellular senescence (Figure S6C,D). In addition, the mRNA expression levels of IL‐6 and IL‐8 were significantly increased following PTCHD4 overexpression (Figure S6E,F). Together, these gain‐ and loss‐of‐function results demonstrate that PTCHD4 plays a crucial regulatory role in promoting cellular senescence across both fibroblasts and alveolar epithelial cells.

### 2.3 PTCHD4 Deficiency Is Associated With Improved Physical Performance in Aging‐Related Mouse Models

Given that PTCHD4 is involved in promoting cellular senescence and is significantly upregulated in aged mouse tissues, we next investigated whether genetic deletion of PTCHD4 could improve physiological functions and delay aging in D‐galactose‐induced aging‐like mice.

**FIGURE 4:**
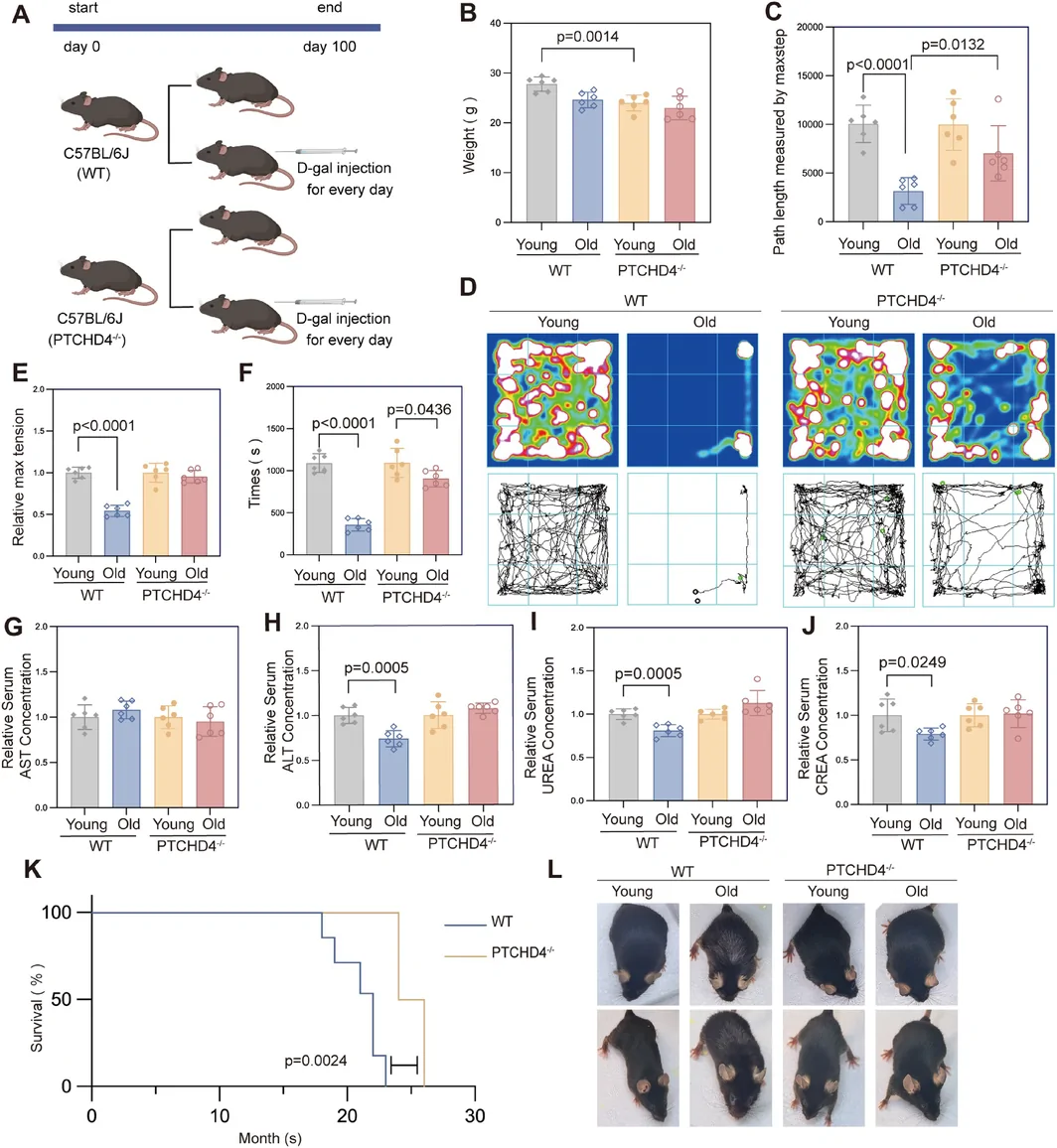
PTCHD4 deficiency is associated with improved physical performance in an aging‐like mouse model. (A) Schematic illustration of the experimental design for the D‐gal‐induced aging model. Twenty‐four 8‐week‐old C57BL/6J mice were randomly separated into four groups: WT, WT D‐gal, PTCHD4^−/−^ and PTCHD4^−/−^ D‐gal. WT or PTCHD4^−/−^ mice in WT or PTCHD4^−/−^ group were injected with daily intervention, while mice in WT D‐gal and PTCHD4^−/−^ D‐gal were injected with D‐gal daily during the experiment period. On day 100, after measuring the physical function, all the mice were sacrificed and their serum was collected. (B) Body weight of WT and PTCHD4^−/−^ mice in young and old groups. (C) Locomotor activity as measured by total path length in the open field test. (D) Representative heatmaps and trajectory plots from the open field test for each group. (E) Relative grip strength (peak force) in young and old WT and PTCHD4^−/−^ mice. (F) Rotarod latency to fall (s) in young and old WT and PTCHD4^−/−^ mice. (G, H) Serum concentrations of AST and ALT. (I, J) Serum concentrations of UREA and CREA. (K) Kaplan–Meier survival curves of WT and PTCHD4^−/−^ mice. Survival differences were analyzed using the log‐rank test (*n* = 6). (L) Representative gross morphological appearance of young and old WT and PTCHD4^−/−^ mice.

To assess the physiological impact of PTCHD4 deficiency, a series of behavioral and cognitive assays were carried out. Both WT and PTCHD4^−/−^ mice were induced to biological aging by intraperitoneal injection of D‐galactose (D‐gal) (Figure 4A), a well‐established model that mimics several physiological characteristics of natural aging (Yang et al. 2023; Ming et al. 2025). Body weight analysis revealed no significant differences in weight gain between genotypes following D‐gal treatment, compared to saline‐treated controls (Figure 4B), indicating no apparent systemic toxicity at the D‐gal dosage used. Our data showed that aged WT mice exhibited markedly reduced locomotor activity and travel distance in the maze test (Figure 4C,D), while PTCHD4^−/−^ mice exhibited substantially higher activity levels. Similarly, PTCHD4^−/−^ mice displayed significantly greater grip strength (Figure 4E; Figure S7) and longer rotarod latency compared with WT controls in this cohort (Figure 4F). These findings suggest that PTCHD4 deficiency may be associated with improved physical performance in the D‐galactose‐induced aging‐like model. Hematoxylin and eosin (HE) staining and Masson's trichrome staining showed no evidence of tissue dysfunction (Figures S8 and S9). Serum biochemical analysis revealed no significant differences in the baseline activities of aspartate transaminase (AST), alanine transaminase (ALT), urea (UREA), and creatinine (CREA) between WT and PTCHD4^−/−^ mice (Figure 4G–J), suggesting that PTCHD4 deficiency does not cause detectable biochemical impairments.

In the subsequent analysis, the impact of PTCHD4 deficiency on lifespan was investigated. The results showed that PTCHD4^−/−^ mice exhibited a significant 25% increase in median lifespan compared to WT controls, with a rise from 20 to 25 months under natural aging conditions (Figure 4K). Our analysis revealed no marked differences in coat conditions among PTCHD4 genotypes. Notably, white hair emergence was observed in aged WT mice. This gross observation was noted but was not further quantified (Figure 4L). These data suggest a possible association between PTCHD4 deficiency and increased survival in this mouse cohort.

### 2.4 PTCHD4 Deficiency Alleviates Bleomycin‐Induced Pulmonary Fibrosis and Improves Lung Function in Mice

**FIGURE 5:**
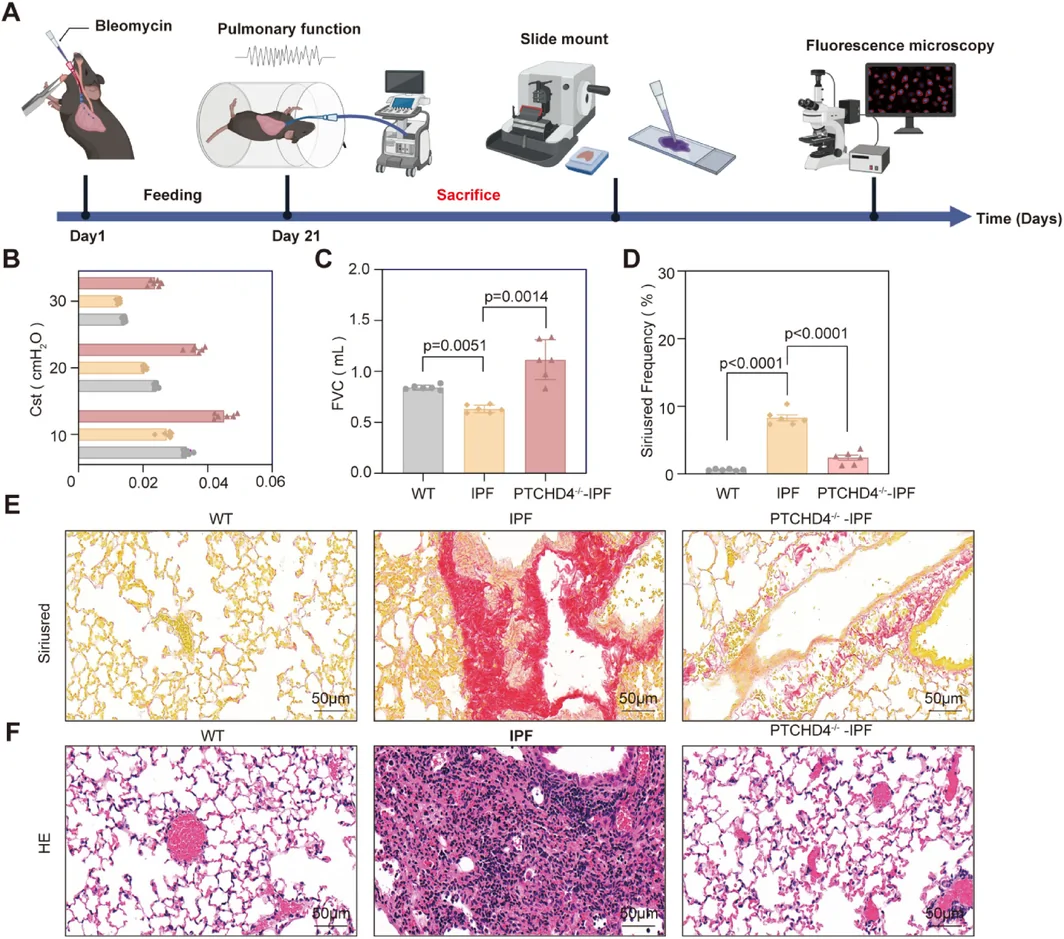
PTCHD4 deficiency alleviates bleomycin‐induced pulmonary fibrosis. (A) Schematic diagram of the bleomycin‐induced pulmonary fibrosis model and experimental timeline. WT mice were maintained under normal conditions. IPF denotes WT mice receiving bleomycin, and PTCHD4^−/−^‐IPF denotes PTCHD4^−/−^ mice receiving bleomycin. Bleomycin was administered on day 0. On day 21, mice underwent pulmonary function tests and were then sacrificed for lung collection and histological analyses (*n* = 6). (B) Static lung compliance (Cst, cmH_2_O) in each group. (C) Forced vital capacity (FVC, mL) in each group. (D) Quantification of fibrosis burden as Sirius Red‐positive area (%) in each group. (E) Representative images of Sirius Red staining. Scale bars, 50 μm. (F) Representative images of H&E staining. Scale bars, 50 μm.

Cellular senescence is involved not only in organismal aging but also in a variety of age‐related diseases. In this study, we employed idiopathic pulmonary fibrosis (IPF), a prototypical age‐associated disorder that predominantly affects individuals over 50 years old (Xu et al. 2025; Raghu et al. 2011) in humans. There are two main reasons for IPF as a model for age‐related disease in this study. Firstly, it allows for direct modeling on PTCHD4 deficiency mice. Secondly, previous research has implicated cellular senescence as a key driver of pulmonary fibrosis, with senescent cell clearance shown to mitigate disease progression (Baker et al. 2011; Xu et al. 2018). For this purpose, WT and PTCHD4^−/−^ mice were intratracheally administered bleomycin (3 mg/kg) and pulmonary fibrosis was established after 21 days. Pulmonary function and histopathological alterations in the lungs were then evaluated (Figure 5A). Pulmonary function testing revealed that forced vital capacity (FVC), an indicator of lung volume and overall compliance, and static lung compliance (Cst), which reflects the elastic recoil and distensibility of lung tissue, were markedly reduced in WT mice at 21 days post induction. However, PTCHD4^−/−^ mice exhibited significantly improved FVC and Cst values under the same conditions (Figure 5B,C). Sirius Red staining, which detects collagen deposition (mainly type I and type III collagen) as an indicator of fibrosis severity, further confirmed that the degree of fibrosis in PTCHD4^−/−^ lungs was substantially lower than in WT controls (Figure 5D,E). Histological examination also revealed that PTCHD4^−/−^ mice exhibited alleviated alveolar structural destruction and reduced inflammatory cell infiltration compared to WT mice after bleomycin treatment (Figure 5F; Figure S10).

Consistent with these findings, immunofluorescence staining further showed pronounced fibrotic remodeling in bleomycin‐treated WT mice, as evidenced by increased COL1A1 and α‐SMA‐positive fibrotic areas around CD31‐positive vascular structures. In contrast, these fibrotic changes were markedly attenuated in bleomycin‐treated PTCHD4^−/−^ mice (Figure S11A). Moreover, co‐staining for SFTPC and p21 revealed an increase in p21‐positive senescence‐associated signals in the alveolar epithelial compartment of WT fibrotic lungs, whereas this increase was reduced in PTCHD4^−/−^ mice (Figure S11B). In line with this observation, the bleomycin‐induced upregulation of IL‐6 and Cxcl2 was significantly suppressed by PTCHD4 deficiency (Figure S11C,D). Additional α‐SMA/p21 co‐immunostaining further indicated that p21‐positive signals were enriched in α‐SMA‐positive regions after bleomycin treatment, and this senescence‐associated alteration was alleviated in PTCHD4^−/−^ mice (Figure S11E).

Collectively, these results indicate that PTCHD4 deficiency not only reduces collagen deposition and fibrotic tissue remodeling but also suppresses senescence‐associated and inflammatory responses in bleomycin‐induced pulmonary fibrosis, thereby improving lung function. These findings support PTCHD4 as a potential therapeutic target for IPF and other senescence‐associated fibrotic diseases.

### 2.5 AKT Signaling Contributes to PTCHD4‐Associated Cellular Senescence

**FIGURE 6:**
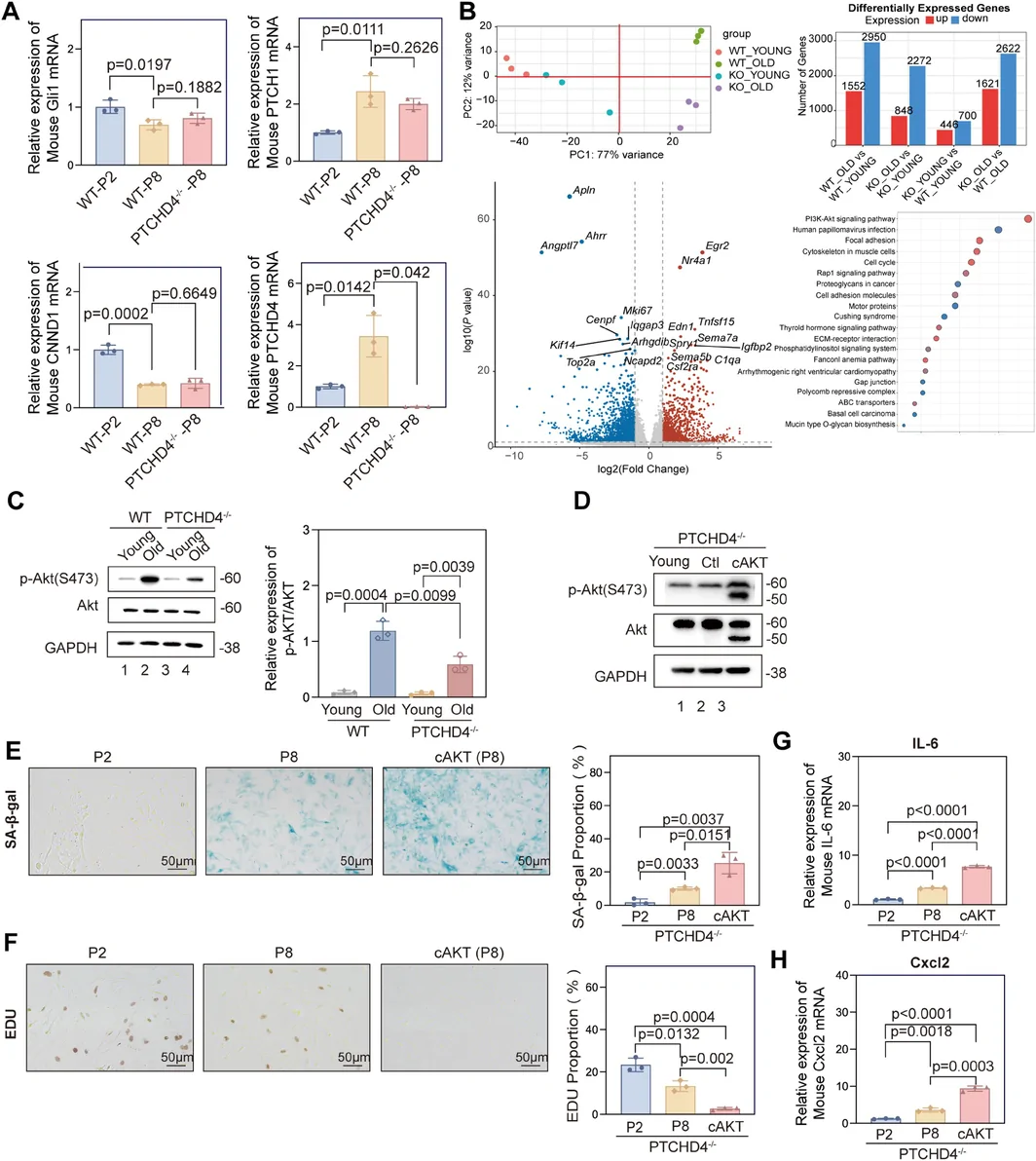
PTCHD4 regulates cellular senescence through AKT signaling. (A) RT‐qPCR analysis of Gli1, PTCH1, PTCHD4 and CCND1 mRNA expression in WT‐P2, WT‐P8 and PTCHD4^−/−^‐P8 (*n* = 3). (B) Transcriptomic profiling of WT and PTCHD4^−/−^ MEFs. PCA shows global separation of WT‐Young, WT‐Old, KO‐Young, and KO‐Old transcriptomes. Volcano plot and bar plot summarize the differentially expressed genes identified in the KO‐Old compared to WT‐Old. KEGG pathway enrichment was performed using differentially expressed genes that were downregulated in the KO‐Old compared to WT‐Old (DESeq2; adjusted *p* < 0.05 and |log2FC| > 1; *n* = 3). (C) Representative images and quantification of protein expression level of Akt, p‐Akt (S473) in WT‐Young, WT‐Old, PTCHD4^−/−^‐Young and PTCHD4^−/−^‐Old. (D) Representative images and quantification of protein expression level of Akt and p‐Akt (S473) in PTCHD4^−/−^‐Young (PTCHD4^−/−^ MEFs at passage 2), PTCHD4^−/−^ +Ctl (PTCHD4^−/−^ MEFs at passage 8), PTCHD4^−/−^ +cAKT (PTCHD4^−/−^ MEFs transfected by cAkt plasmid at passage 2) (*n* = 3). (E, F) Representative images and quantification of SA‐β gal and EdU staining in PTCHD4^−/−^‐P2, PTCHD4^−/−^‐P8 and PTCHD4^−/−^ +cAKT (*n* = 3). (G, H) mRNA expression of IL‐6 and Cxcl2 in group PTCHD4^−/−^‐P2, PTCHD4^−/−^‐P8 and PTCHD4^−/−^ +cAKT (*n* = 3).

As a homolog of PTCH1, a known inhibitor of the Hedgehog (Hh) signaling pathway, we initially examined whether PTCHD4 similarly modulates Hh signaling pathway. However, the expressions of the Hh target genes such as Gli1, PTCH1, and CCND1 (Cyclin D1) showed no significant change when PTCHD4 was manipulated (Figure 6A). These data suggest that canonical Hh target gene activation is unlikely to explain PTCHD4‐associated senescence under these conditions.

To elucidate the mechanism by which PTCHD4 promotes senescence, we performed Transcriptome sequencing on four distinct groups of MEFs, including young WT, old WT, young PTCHD4^−/−^ and old PTCHD4^−/−^. Principal component analysis (PCA) revealed tight intra‐group clustering of transcriptomic profiles and clear separation among the four groups. Differential gene expression analysis identified 1621 upregulated and 2622 downregulated genes in old PTCHD4^−/−^ MEFs compared to old WT MEFs. Notably, KEGG enrichment analysis (KEGG) indicated significant downregulation of the PI3K‐AKT signaling pathway in the old PTCHD4^−/−^ group (Figure 6B).

We next assessed AKT pathway activation levels between the four groups. Phospho‐AKT (S473) levels were significantly elevated in old MEFs compared to young controls. A similar increase was observed in old PTCHD4^−/−^ MEFs relative to young PTCHD4^−/−^ MEFs. However, the extent of AKT phosphorylation in old PTCHD4^−/−^ MEFs was markedly lower than in old WT MEFs, suggesting that PTCHD4 deficiency attenuates aging‐associated AKT activation (Figure 6C). To further investigate the functional role of the AKT pathway in PTCHD4‐mediated senescence, we stably expressed a constitutively active AKT construct (cAkt) in PTCHD4^−/−^ MEFs (Figure 6D). Restoration of AKT signaling significantly reversed the anti‐senescence effects of PTCHD4 deficiency, as evidenced by reduced EdU incorporation, increased SA‐β gal activity, expression of SASP factors and senescence markers, indicating the reappearance of the senescence phenotype (Figure 6E–H). Through complementation experiments, we introduced a dominant‐negative AKT mutant (AktDN), which can be phosphorylated but lacks kinase activity and competitively inhibits endogenous AKT signaling, into young WT MEFs. Stable expression of AktDN significantly reduced endogenous AKT signaling (Figure S12A). This inhibition of AKT signaling led to a direct attenuation of SASP expression, SA‐β gal activity and restored EdU incorporation (Figure S12B–E). This effect was comparable to treatment with the AKT inhibitor MK‐2206 (Figure S12B–E).

Given the relevance of AEC2s to pulmonary fibrosis and epithelial senescence, we further examined whether AKT activation was also associated with senescence‐inducing stimuli and PTCHD4 expression in AEC2s. Both irradiation and bleomycin treatment increased phospho‐AKT levels in AEC2s (Figure S13A). Moreover, PTCHD4 overexpression was sufficient to significantly enhance AKT phosphorylation in AEC2s (Figure S13B). These results extend the association between PTCHD4 and AKT activation from MEFs to lung epithelial cells. PTCHD4‐FLAG pull‐down followed by DIA‐MS did not show selective enrichment of AKT1/2/3 or major AKT pathway‐related proteins in AEC2 or HUVEC cells, suggesting that PTCHD4 is unlikely to regulate AKT signaling through a stable direct physical interaction under the tested conditions (Figure S14).

**FIGURE 7:**
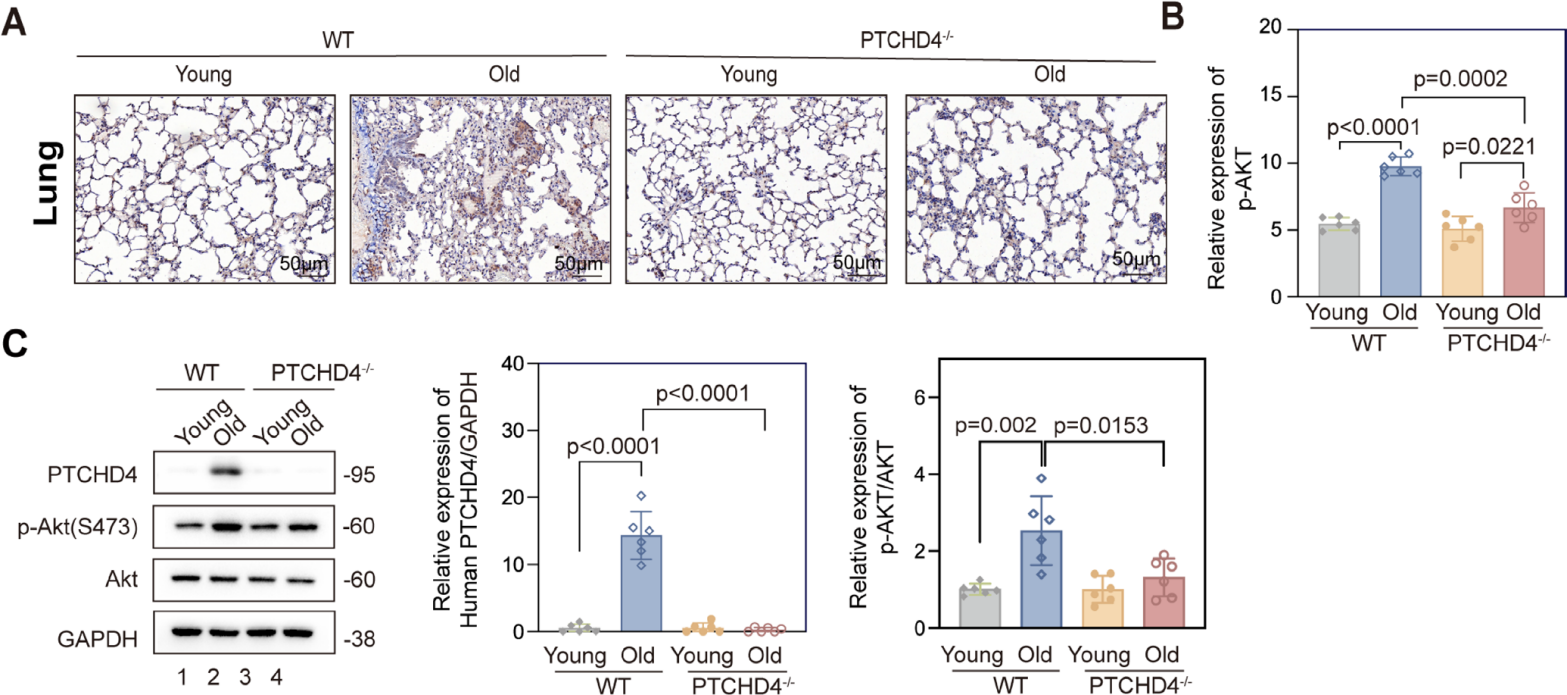
PTCHD4 deficiency attenuates AKT activation in aged cells. (A) Representative IHC images showing phosphorylated AKT (p‐AKT, Ser473) staining in lung sections from young and old WT and PTCHD4^−/−^ mice. Scale bar, 50 μm. (B) Quantification of p‐AKT (Ser473) IHC staining. Data are presented as mean ± SEM (*n* = 6). (C) Representative Western blot images and quantification of phosphorylated AKT (Ser473), total AKT, and PTCHD4 in young and old WT and PTCHD4^−/−^ mice (*n* = 6).

Finally, to further validate the relevance of PTCHD4 expression to the activity of the Akt signal pathway in vivo, we assessed p‐AKT expression in lung tissue from young WT, aged (D‐gal‐induced) WT and PTCHD4^−/−^ mice via IHC (Figure 7A,B) staining and lung tissue Western blot (Figure 7C). The results showed that in WT animals, D‐gal‐induced aging significantly increased PTCHD4 expression, which was accompanied by elevated p‐AKT levels, demonstrating enhanced AKT activation in response to aging. Notably, PTCHD4^−/−^ mice exhibited a similar increase in p‐AKT expression relative to their WT counterparts, supporting the role of PTCHD4 in promoting aging‐associated AKT activation.

## 3 Discussion

PTCHD4 belongs to the patched domain protein family. Although it was initially annotated as a putative membrane protein with largely undefined biological functions, emerging evidence now implicates PTCHD4 in fundamental cellular processes, including cancer biology and stress responses (Malatesta et al. 2024; Rossi et al. 2024). Given its structural similarity to PTCH1 and its responsiveness to cellular stress, PTCHD4 has recently attracted attention as a potential regulator of age‐related signaling networks (Esmaeli et al. 2025). However, its physiological relevance and mechanistic contribution to these processes remain poorly understood.

In this study, we identified PTCHD4 as a senescence‐associated transmembrane protein that contributes to cellular senescence and age‐associated phenotypes. PTCHD4 expression was increased across multiple senescence models and aged mouse tissues, whereas PTCHD4 deficiency attenuated senescence‐associated phenotypes in vitro and was associated with improved physical performance and extended median lifespan in mice. These findings suggest that PTCHD4 may represent a candidate target for senescence‐associated interventions. Our findings further support a potential role for PTCHD4 in age‐related fibrotic disease. In the bleomycin‐induced pulmonary fibrosis model, PTCHD4 deficiency reduced collagen deposition, attenuated inflammatory and senescence‐associated signals, and improved pulmonary function. Importantly, PTCHD4 was upregulated not only in fibroblasts but also in AEC2 cells during senescence induction, and PTCHD4 overexpression promoted senescence‐associated phenotypes in these cells. Together with analyses of public human lung datasets, which revealed enrichment of PTCHD4‐positive epithelial populations during senescence, including AT2 cells and aberrant basaloid cells. These results suggest that PTCHD4 is associated with age‐related lung remodeling and fibrotic pathology.

The induction of PTCHD4 during senescence may involve multiple regulatory mechanisms. p53 is a key regulator of cell‐cycle arrest and DNA damage responses during cellular senescence. It has been shown that p53 positively regulates PTCHD4 expression at the transcriptional level (Chung et al. 2014), suggesting that PTCHD4 is involved in a well‐established senescence‐induced pathway. A recent study reported that PTCHD4 expression is also regulated at the post‐transcriptional level by METTL3/METTL14‐mediated m6A modification and IGF2BP1‐dependent mRNA stabilization in senescent cells (Rossi et al. 2024). Together, these findings suggest that PTCHD4 expression during senescence is controlled by multiple regulatory layers.

Mechanistically, our results indicate that AKT signaling contributes to PTCHD4‐associated senescence. The PI3K‐AKT pathway is a well‐characterized regulator of aging and persistent AKT activation has been shown to accelerate senescence by enhancing mTOR signaling, increasing oxidative stress, and strengthening p53/p21‐ and p16/Rb‐mediated cell‐cycle arrest (Hu et al. 2023; Rivas et al. 2022; Chou et al. 2023). In vivo, AKT hyperactivation is associated with shortened lifespan and heightened susceptibility to age‐related disorders, whereas AKT inhibition can delay senescence and improve metabolic and physiological resilience (Hamwi et al. 2024; Yu et al. 2022; Han and Kim 2025). In this study, our data suggest that PTCHD4 contributes to full AKT activation during senescence. However, further research is needed to identify target proteins through which PTCHD4 regulates the PI3K‐AKT pathway.

While this study establishes PTCHD4 as a critical regulator of cellular senescence and age‐related pathologies through AKT signaling, several limitations should be acknowledged. First, the mechanistic link between PTCHD4 and AKT activation remains incompletely defined. Further research is required to identify key intermediate molecules and clarify the precise molecular cascade connecting PTCHD4 to AKT activation. Second, although public human transcriptomic datasets support the relevance of PTCHD4 in aging and its‐associated lung fibrosis, direct evidence from primary human tissue samples is absent. Validation in human clinical specimens will be essential to strengthen the rationale for targeting PTCHD4 in clinical interventions against age‐related disorders. Third, PTCHD4 remains a poorly characterized transmembrane protein, and the current analyses do not exclude subtle, long‐term, organ‐specific, or context‐dependent effects. Future studies will require systematic safety assessments, including organ‐specific functional assays, reproductive assessment, and evaluation under additional physiological or pathological stress conditions.

In summary, our study identifies PTCHD4 as a previously unrecognized contributor to cellular senescence and age‐associated tissue dysfunction. By linking PTCHD4 to AKT signaling and pulmonary fibrosis, these findings provide a basis for further investigation of the PTCHD4‐AKT axis as a potential target in senescence‐associated diseases.

## 4 Materials and Methods

### 4.1 Materials

#### 4.1.1 Cell Culture and Cellular Senescence Induction

Dulbecco's Modified Eagle Medium (DMEM, FI101‐01), Fetal Bovine Serum (FBS, FS201‐02) were acquired from TransGEN (Beijing, China). Cascade Biologics Medium (M200500), Advanced DMEM/F‐12 Medium (12634010), Pen Strep (P/S, 15140122) and 0.25% Trypsin–EDTA (25200072) were purchased from Thermo Fisher Scientific (USA).

Mouse embryonic fibroblasts (MEFs) were isolated from 12.5‐day‐old WT or PTCHD4^−/−^ mouse embryos. Briefly, embryos were dissected, and the heads and visceral tissues were removed. The remaining embryonic tissues were minced and digested with 0.25% Trypsin–EDTA for 5–10 min at 37°C. The resulting cell suspension was collected and cultured in DMEM supplemented with 10% FBS and 1% penicillin–streptomycin at 37°C in a humidified atmosphere with 5% CO_2_. MEFs within four passages were considered young cells. Replicative senescence was induced by continuous passaging, and passage 8 MEFs were used as senescent cells unless otherwise indicated.

All cell lines were cultured at 37°C in a humidified atmosphere with 5% CO_2_. MEFs, 2BS, and 293 T cells were maintained in DMEM supplemented with 10% FBS and 1% P/S. HUVECs were cultured in endothelial cell medium containing 5% FBS, 1% endothelial cell growth supplement, and 1% P/S. ARPE‐19 cells were cultured in DMEM/F12 medium supplemented with 10% FBS and 1% P/S.

For MEFs, the senescence model was established using a passage‐dependent approach, with cells being passaged 8 times. MEFs within 4 passages were considered as young cells. MEFs were isolated from 12.5‐day‐old mouse embryos. Briefly, the embryos without viscera and heads were dissected and digested with 0.25% Trypsin–EDTA for 5–10 min. The single‐cell suspensions were cultured in DMEM medium containing 10% FBS and 100 units/mL Penicillin–Streptomycin at 37°C in a humidified atmosphere with 5% CO_2_.

Cellular senescence was induced using bleomycin‐induced premature senescence; cells were treated with 50 μg/mL bleomycin for 24 h. Following treatment, complete culture medium was added, and the medium was replaced every 2 days for 7 days.

#### 4.1.2 Plasmid Construction, Transient Transfection and Pharmacological Inhibition

Plasmids. The full‐length coding sequence (CDS) of mouse PTCHD4 was cloned into the PITA vector, with the PITA vector being used as the negative control. The constitutively active AKT (cAkt1) and the dominant‐negative Akt1 (AktDN) constructs were obtained from Addgene (pHRIG‐Akt1, plasmid #53583 and pHRIG‐AktDN, plasmid #53597; depositor: Heng Zhao). All plasmids were verified by sequencing (Sanger, China).

Transient transfection. MEFs were seeded 16–24 h prior to transfection to achieve 60%–80% confluence. Transient transfections were performed using Lipofectamine 3000 (Invitrogen, USA) according to the manufacturer's instructions. For each well of a 6‐well plate, 5 μg plasmid DNA (Ptchd4, cAkt, AktDN or Ctl vector) was used. The medium was replaced 6 h post‐transfection, and cells were harvested 48 h after transfection for subsequent assays. Pharmacological inhibition of AKT. MEFs were treated with MK‐2206 to inhibit AKT signaling (TargetMol Chemicals Inc., China).

#### 4.1.3 Animals

All animal experiments were approved by the Institutional Animal Care and Use Committee of Peking University Health Science Center (Approval Number: DLASBE0201).

All animal experiments were performed in accordance with the animal welfare guidelines of Peking University Health Science Center, China. Mice were housed in a specific pathogen‐free (SPF) facility under controlled conditions, with a 12‐h light/dark cycle and provided free access to food and water.

For physical function measurements, twenty‐four 8‐week‐old C57BL/6J male mice (purchased from Peking University Health Science Center, China) were randomly divided into four groups: WT, WT + D‐gal, PTCHD4^−/−^ and PTCHD4^−/−^ + D‐gal. Mice in the WT and PTCHD4^−/−^ groups were fed without any intervention, while those in WT D‐gal and PTCHD4^−/−^ D‐gal were injected with D‐galactose daily throughout the experimental period. On day 100, physical function was assessed and all mice were sacrificed. Blood samples were then collected for serum analysis.

For pulmonary function measurements, the mice were randomly assigned to experimental groups. Pulmonary fibrosis was induced by a single intratracheal instillation of bleomycin (3 mg/kg; dissolved in sterile saline) on day 0 under anesthesia. Control mice received an equal volume of sterile saline. Mice were monitored daily, and their lungs were harvested on day 21 for histological analyses (HE, Masson's trichrome/Sirius Red, as applicable) and molecular assays. Investigators were blinded to group allocation during outcome assessment.

For tissue aging measurements, the mice were divided into three groups: young, old, and progeroid mice. Young WT mice were sacrificed at 8 weeks, and old WT mice were sacrificed at 90 weeks. The progeroid group consisted of Zmpste24^−/−^ that was sacrificed at 12 weeks. All mice were on a C57BL/6J background. For each group, six male mice were used for tissue collection and IHC analysis.

### 4.2 Physical Function Measurements

All functional measurements were conducted at least 5 days after the final D‐gal injection. The mice were trained on days 1 and 2, and tested on days 3, 4, and 5. The results showed the average of three trials.

Motor coordination and balance were assessed using a rotarod apparatus (Unibiolab). The test protocol was configured with an initial speed of 5 rpm, which accelerated at a rate of 0.1 rpm/s up to a maximum of 30 rpm. The trial was terminated either when the mouse fell from the rod or upon reaching the maximum predefined duration of 600 s. The device automatically documented the latency to fall (in seconds) and the corresponding rotational speed (in rpm) at the time of fall.

Grip strength was assessed using a grip strength meter (Unibiolab). Each mouse was allowed to securely grasp the device's mesh or wire grid with all four limbs. Subsequently, the mouse was pulled backward gently by the tail in a horizontal plane until it released the grid. The trial was repeated five times for each animal, and both the maximum and average grip strength (in Newtons, N) were automatically recorded for analysis.

Locomotor activity was assessed using an open field apparatus (Unibiolab). Mice were acclimated to the apparatus for 30 min prior to testing. Subsequently, each mouse was placed individually in a 40 cm × 40 cm square arena and its behavior was recorded for 10 min. A camera system tracked locomotor activity, including travel trajectories and speed. The test was performed under bright, quiet conditions, with the apparatus cleaned between trials to eliminate potential olfactory biases.

### 4.3 Lifespan Analysis

WT and PTCHD4^−/−^ mice were housed under SPF conditions in a 12‐h light/dark cycle. The mice were monitored from weaning until natural death. The health of the mice was observed daily. Survival time was defined as the age at death. Survival curves were generated using the Kaplan–Meier method and compared using the log‐rank test.

### 4.4 Blood Analysis

Serum biochemical parameters were analyzed from collected blood samples. The samples were allowed to clot at 37°C for 45 min, followed by overnight incubation at 4°C. Subsequently, the samples were centrifuged at 1000 g for 10 min to isolate the serum. Aliquots of 200 μL of the resulting serum were used to measure the levels of alanine aminotransferase ALT, AST, UREA and CREA using a Mindray Chemray 800 chemical analyzer.

### 4.5 Pulmonary Function Measurement

Invasive pulmonary function tests were performed in anesthetized mice using a computer‐controlled small‐animal ventilator (AniRes2005, Peking BioLab Tech, China). Mice were intubated and mechanically ventilated under standardized settings. FVC was obtained using a negative pressure‐driven forced expiration (NPFE) maneuver: lungs were inflated to a predefined pressure approaching TLC (30 cmH_2_O) followed by rapid switching to a negative pressure reservoir to generate a forced expiratory flow‐volume curve, from which FVC was calculated. Static compliance (Cst) was derived from quasi‐static pressure‐volume (P‐V) loops generated by stepwise inflation and deflation with brief end‐inspiratory/expiratory holds (zero‐flow conditions). The slope of the P‐V relationship within the predefined linear range was used to calculate Cst.

### 4.6 Sirius Red Staining

Lung tissue sections from mice were deparaffinized and rehydrated through a graded ethanol series. The sections were then stained with Sirius Red staining solution for 15 min to visualize collagen fibers. After a brief rinse in distilled water to remove excess dye, the sections were dehydrated in graded ethanol, cleared in xylene and mounted with neutral balsam.

### 4.7 HE and Masson's Trichrome Staining

HE staining and Masson's trichrome staining were performed on paraffin‐embedded sections to assess tissue morphology and collagen deposition. Briefly, tissues (lung, liver, kidney, and heart) were fixed, paraffin‐embedded and sectioned at 5 μm. The sections were then deparaffinized and rehydrated, followed by HE staining (G1076‐500ML, Servicebio, China) or Masson's trichrome staining (G1006‐20ML, Servicebio, China) using a commercial kit according to the manufacturer's instructions. Bright‐field images were acquired using a light microscope (Zeiss axioscan 7, Zeiss, Germany) under identical settings for all groups. When applicable, the positive area was quantified in a blinded manner using ImageJ.

### 4.8 Immunohistochemistry

The lung, liver, kidney and heart tissues were fixed in 4% paraformaldehyde at room temperature for 48 h. Following paraffin embedding, the tissues were sliced into 5 μm sections and subjected to immunohistochemical staining. Briefly, the sections were incubated with antibodies specific for PTCHD4 (NBP1‐84223, NOVUS), P16 (ab54210, abcam), phospho‐AKT (Ser473) (#9271, CST) at 4°C overnight, followed by staining with secondary antibodies. The sections were then stained with horseradish peroxidase (HRP)‐conjugated streptavidin and DAB solution.

### 4.9 Western Blot

Cells samples were lysed by RIPA buffer (899,001, Thermo Fisher Scientific, USA) containing protease and phosphatase inhibitors (Pierce Phosphatase Inhibitor Mini Tablets, a32961, Thermo Fisher Scientific, USA) on ice for 30 min. The lysates were centrifuged at 12,000 rpm for 15 min at 4°C. Protein concentrations were determined using a The Pierce BCA Protein Assay Kit (23,227, Thermo Fisher Scientific, USA). An equal amounts of protein were mixed with 2 × SDS loading buffer, boiled at 100°C for 5 min, and separated by SDS‐PAGE using 7.5%, 10%, or 12.5% polyacrylamide gels. The gel electrophoresis was performed at 80 V for 20 min and 120 V for 40 min. Proteins were transferred onto PVDF membranes (Immobilon ‐PSQ PVDF Membrane, ISEQ00010, Millipore, USA) at 400 mA for 20 to 90 min, depending on the molecular weight of the protein. The membranes were blocked with 5% BSA in TBST for 1 h at room temperature. After blocking, the membranes were incubated overnight at 4°C with primary antibodies. The membranes were then incubated with HRP‐conjugated secondary antibodies for 1 h at room temperature. Protein signals were detected using a ChemiDoc imaging system (Bio‐Rad, USA). The band intensity was quantified using ImageJ software (National Institutes of Health, USA). Primary antibodies were listed as follow (Table S1).

### 4.10 RT‐qPCR

Total RNA was extracted from cells with TransZol Up reagent (ET111‐01, TransGEN, Beijing, China) and reversed into cDNA by TransScript II All‐in‐One First Strand cDNA Synthesis Supermix (AH341‐0, TransGEN, Beijing, China). Quantification of cDNA was performed using RT‐qPCR with PerfectStart Green qPCR Supermix (AQ602‐01, TransGEN, Beijing, China). RT‐qPCR was performed using the 7500 Fluorescence quantitative PCR System (ABI, USA). Gene expression changes were calculated using the ∆∆CT method, with GAPDH serving as the endogenous normalization control. The primers used as follows (Table S2).

### 4.11 SA‐β Gal **Staining**

Fresh adherent cells were washed twice by cold PBS and fixed with 4% paraformaldehyde for 5–10 min at room temperature. After two cold PBS washes, the samples were incubated with freshly prepared SA‐β gal staining solution (containing 1 mM MgCl_2_, 5 mM potassium ferricyanide, 5 mM potassium ferrrocynide and 1 mg/mL X‐gal, pH = 6) overnight at 37°C. PBS was used to end the staining for three times. The photography was captured by digital microscope image system (Leica, Germany).

### 4.12 EdU Cell Proliferation Detection

The cells were cultured in a 6 cm plate and incubated overnight to allow for adhesion. A 2 × EdU working solution (20 μM) was prepared before conducting the experiment. An equal volume of the 2 × EdU working solution was added to the cell cultures, which were then incubated at 37°C for 2 h. Following EdU labeling, the culture medium was removed, and the cells were fixed. The cells were washed three times with PBS, then incubated with an endogenous peroxidase blocking solution at room temperature for 20 min. The click reaction mixture was added to the plate, and the cells were incubated in the dark at room temperature for 30 min. The Streptavidin‐HRP working solution and DAB chromogen solution were prepared and sequentially added to the plate. After incubation for 30 min with each solution, the chromogen reaction was washed, and images were captured using a digital microscope system (Leica, Germany).

### 4.13 Transcriptome Sequencing

MEFs from four groups‐young WT (WT‐Y), old WT (WT‐O), young Ptchd4^−/−^ (KO‐Y) and old Ptchd4^−/−^ (KO‐O) were collected for transcriptome profiling (*n* = 3). Young and old MEFs were defined as passage P2 and P8. Total RNA was extracted from MEFs using TRIzol reagent (Sigma, USA). RNA concentration was measured using a NanoDrop Spectrophotometer (Thermo Fisher Scientific, USA), and samples with RNA integrity number (RIN) ≥ 7.0 and 28S/18S > 1.8 were used for library preparation. The libraries were prepared and sequenced using a NovaSeq 6000 (Illumina, USA). Transcriptome sequencing were conducted by Shanghai Applied Protein Technology Co. Ltd.

### 4.14 Bioinformatic Analysis

Raw paired‐end sequencing reads were initially processed using cutadapt (Kechin et al. 2017) for adapter trimming and preliminary quality filtering. Illumina adapter sequences were explicitly specified for forward (AGATCGGAAGAGCACACGTCTGAACTCCAGTCAC) and reverse (AGATCGGAAGAGCGTCGTGTAGGGAAAGAGTGTAGATCTCGGTGGTCGCCGTATCATT) reads. Reads shorter than 15 bp, containing more than eight ambiguous nucleotides, or failing paired‐end consistency filtering were removed (‐‐minimum‐length 15, ‐‐max‐n 8, ‐‐pair‐filter = any). Adapter‐trimmed read pairs were further subjected to quality filtering using Trimmomatic (Bolger et al. 2014) in paired‐end mode with Phred+33 encoding. Filtering criteria included an average read quality threshold of 20 (AVGQUAL:20), sliding window trimming with a 4‐bp window and a minimum average quality of 15 (SLIDINGWINDOW:4:15), and a minimum read length of 15 bp (MINLEN:15). High‐quality reads were aligned to the mouse reference genome (mm10, GRCm38) using HISAT2 (Kim et al. 2019) with default parameters. Resulting SAM files were converted to BAM format, followed by sorting and indexing using SAMtools (Li et al. 2009). Gene‐level read counts were generated using featureCounts (Liao et al. 2014), assigning reads to exonic regions (−t exon) and summarizing counts at the gene level based on gene_id annotations from the Ensembl Mus_musculus GRCm38.102 GTF file. Differential gene expression analysis was performed using DESeq2 (Love et al. 2014). Principal component analysis (PCA) was conducted based on variance‐stabilized transformed expression values to assess global transcriptional differences among samples. Differentially expressed genes were identified using the DESeq2 statistical framework, with genes showing an adjusted *p* < 0.05 and an absolute log2(fold change) greater than 1 considered statistically significant. Functional enrichment analysis of differentially expressed genes was performed using clusterProfiler (Yu and He 2012). Gene Ontology (GO) and Kyoto Encyclopedia of Genes and Genomes (KEGG) pathway enrichment analyses were conducted to identify significantly overrepresented biological processes and pathways.

### 4.15 Mice Breeding and Genotyping

To generate PTCHD4^−/−^ mice, PTCHD4^+/−^ mice were intercrossed. Offspring from subsequent litters were ear‐notched or tail‐tipped for genotyping. Genomic DNA was isolated from mouse tail clips (approximately 0.3–0.5 cm) using a Quick Genotyping Assay Kit (D7283M, Beyotime, China). Genotyping was performed by multiplex PCR using two pairs of primers (Table S3).

PCR was performed using a standard reaction system and cycling conditions. The genotypes were determined by analyzing the PCR products on an agarose gel. The genotyping criteria were as follows: WT: A single 294 bp band from P3/P4. Heterozygous (HE): Both the 673 bp (P1/P2) and 294 bp (P3/P4) bands. Homozygous (HO): A single 673 bp band from P1/P2. Through this breeding strategy and genotyping protocol, PTCHD4^−/−^ mice were successfully generated for subsequent experiments.

### 4.16 Public Human Transcriptomic Dataset Analysis

Public human lung transcriptomic datasets were re‐analyzed to assess the association of PTCHD4 with human lung aging and fibrotic lung disease. For scRNA‐seq datasets, analyses were performed at the sample or subject level to avoid pseudoreplication. AT2/ATII epithelial cells and aberrant basaloid cells were annotated according to the original dataset‐specific analyses. Because PTCHD4 is a low‐abundance transcript in scRNA‐seq data, PTCHD4 positivity was defined as detectable expression greater than zero, and the primary endpoint was the proportion of PTCHD4‐positive cells within each sample or subject.

For IPF‐related analyses, PTCHD4‐positive proportions in AT2/ATII cells were compared between control and IPF samples using Wilcoxon rank‐sum tests. Aberrant basaloid cells in GSE136831 were summarized descriptively because this population was absent or nearly absent in control lungs. For aging analyses, associations between PTCHD4 expression or PTCHD4‐positive AT2 cell percentage and donor age were assessed using Spearman correlation. Differences across age groups were evaluated using Kruskal‐Wallis tests.

To explore whether PTCHD4‐positive epithelial cells were associated with senescence‐related transcriptional features, we performed transcript‐level co‐detection analyses in scRNA‐seq datasets with available raw count data or final Seurat objects. Senescence/SASP‐associated markers included CDKN1A, CDKN2A, TP53, SERPINE1, IL6, CXCL8, MMP7, MMP9 and MMP14. Within each sample or subject, senescence marker module scores were calculated separately for PTCHD4‐positive and PTCHD4‐negative epithelial cells, and paired sample‐level differences were tested using Wilcoxon signed‐rank tests. These analyses were interpreted as transcript‐level co‐detection or enrichment analyses and not as spatial protein co‐localization.

### 4.17 Statistical Analysis

Statistical analyses were performed using GraphPad Prism and R software. Data are presented as mean ± SD. For experimental data, two‐tailed unpaired Student's *t*‐test were used unless otherwise indicated. Survival curves were generated using the Kaplan–Meier method and compared by log‐rank test. Differentially expressed genes in RNA‐seq analysis were identified using DESeq2, with adjusted *p* < 0.05 and |log2 fold change| > 1 considered significant. For public human transcriptomic datasets, Wilcoxon rank‐sum tests, Wilcoxon signed‐rank tests, Spearman correlation analysis, or Kruska‐Wallis tests were used as appropriate. *p* < 0.05 was considered statistically significant. *n* represents independent biological replicates or individual mice in animal experiments.

## Author Contributions

Conceptualization, writing – review and editing, and supervision: Z.M., Y.L., and X.B. Methodology: M.W. and Z.Y. Investigation: Z.L. and Z.H. Data Curation: L.W. and X.S. Formal analysis: D.Y. and W.Y. Writing‐original draft: M.W. and Q.W.

## Funding

This work was supported by National Natural Science Foundation of China (32371533, 82271619, and 82573232).

## Conflicts of Interest

The authors declare no conflicts of interest.

## Supporting information


**Figure S1:** PTCHD4 is upregulated and accompanies cellular senescence in MEFs.
**Figure S2:** Irradiation and bleomycin induce PTCHD4 expression and senescence phenotypes in AEC2.
**Figure S3:** Aging Atlas reveals increased PTCHD4 expression in senescent cells.
**Figure S4:** PTCHD4‐positive epithelial cells are increased in IPF and associated with aging and senescence signatures in human lung datasets.
**Figure S5:** PTCHD4 overexpression accelerates cellular senescence.
**Figure S6:** PTCHD4 overexpression promotes senescence‐associated phenotypes in AEC2 cells.
**Figure S7:** Grip strength assessment in young and old WT and PTCHD4^−/−^ mice.
**Figure S8:** Age‐associated histological changes in multiple organs of WT and PTCHD4^−/−^ mice.
**Figure S9:** Fibrotic features in multiple organs of young and aged WT and PTCHD4^−/−^ mice.
**Figure S10:** PTCHD4 deficiency attenuates pulmonary fibrosis.
**Figure S11:** PTCHD4 deficiency attenuates fibrosis‐associated senescence and inflammation in bleomycin‐induced pulmonary fibrosis.
**Figure S12:** Inhibition of Akt alleviates the cellular senescence.
**Figure S13:** PTCHD4 promotes AKT activation in AEC2 cells.
**Figure S14:** PTCHD4‐FLAG pull‐down/DIA‐MS does not show selective enrichment of AKT family proteins.
**Table S1:** Primary antibody used were listed as follow.
**Table S2:** The primers used were listed as follow.
**Table S3:** Genotyping primers for PTCHD4^−/−^ mice.

## Data Availability

All data are available in the main text or the Supporting Information S1. The high‐throughput sequencing datasets generated in this study have been deposited in the Gene Expression Omnibus (GEO) under accession number GSE314645 (token: anczoqainlmrbor). *Code availability*: The code used for data processing and analysis in this study is publicly available on GitHub at: https://github.com/yudonglin506311858/PTCHD4_KO_PROJECT.
