# Supplementary material for: Transmembrane Protein PTCHD4 Is a Novel Regulator of Cellular Senescence and Age‐Related Pathologies
Source: Aging Cell. 2026 Sep 11;25(9):e70711. doi: 10.1111/acel.70711 (PMC13564249; doi:10.1111/acel.70711)
Supplement: Supplementary file 1 [file ACEL-25-e70711-s001.docx]

Supplementary Materials for

**Transmembrane Protein PTCHD4 Is a Novel Regulator of Cellular Senescence and Age-Related Pathologies**

Mingyue Wang *et al.*

*Corresponding author. Email: [liyang@hsc.pku.edu.cn](mailto:liyang@hsc.pku.edu.cn)

*Corresponding author. Email: zbmao@bjmu.edu.cn

*Corresponding author. Email: baoxiaoxiazw@163.com

**This PDF file includes:**

Supplementary Text

Figs. S1 to S14#

Tables. S1 to S3#

Figure S1 PTCHD4 is upregulated and accompanies cellular senescence in MEFs

(A-B) Representative images and quantification of EdU and SA-β-galactosidase staining in young and old MEFs (n=3). Scale bars, 50 μm. (C) Representative Western blot images and quantification of PTCHD4, p53 and p16^INK4A^ protein levels in young and old MEFs (n=3). (D) RT-qPCR analysis of PTCHD4 mRNA expression in young and old MEFs (n=3). (E-F) RT-qPCR analysis of IL-6 and Cxcl2 mRNA levels in young and old MEFs (n=3).

Figure S2 Irradiation and bleomycin induce PTCHD4 expression and senescence phenotypes in AEC2

(A) Representative Western blot images and quantification of PTCHD4, p53 and p21 protein levels in IR- and Bleo-treated AEC2 cells compared with NC group (n=3). (B) RT-qPCR analysis of PTCHD4 mRNA expression in IR- and Bleo-treated AEC2 cells compared with NC group (n=3). (C-D) Representative images and quantification of EdU and SA-β-galactosidase staining in IR- and Bleo-treated AEC2 cells compared with NC group (n=3). Scale bars, 50 μm. (E-F) RT-qPCR analysis of IL-6 and IL-8 mRNA levels in IR- and Bleo-treated AEC2 cells compared with NC group (n=3).

Figure S3 Aging Atlas reveals increased PTCHD4 expression in senescent cells

A-C, Expression data of PTCHD4 mRNA from Aging Atlas(https://ngdc.cncb.ac.cn/aging/index). (A)Human diploid fibroblasts(WI38). (B)Human diploid fibroblasts(IMR90). (C)Human arterial endothelial cell (HAECs).

Figure S4 PTCHD4-positive epithelial cells are increased in IPF and associated with aging and senescence signatures in human lung datasets.

(A-C) Analysis of public human lung single-cell transcriptomic datasets showing increased proportions of PTCHD4-positive AT2/ATII cells in IPF samples compared with controls. (D) Quantification of aberrant basaloid cells among ATII and aberrant basaloid epithelial cells in the GSE136831 dataset, showing enrichment of aberrant basaloid cells in IPF lungs. (E-F) Analysis of the GSE165192 dataset showing that PTCHD4 expression positively correlates with age and differs across age groups. (G-H) Analysis of the GSE281219 dataset showing an age-associated increase in the proportion of PTCHD4-positive AT2 cells. (I) Subject-level paired analysis in IPF aberrant basaloid cells showing enrichment of senescence-related markers in PTCHD4-positive cells compared with PTCHD4-negative cells. (J-K) Paired analysis of senescence/SASP module scores in PTCHD4-positive versus PTCHD4-negative epithelial cells from the indicated datasets, showing higher senescence/SASP signatures in PTCHD4-positive cells. Statistical methods and *p* values are indicated in each panel.

Figure S5 PTCHD4 overexpression accelerates cellular senescence

(A) Representative Western blot images and quantification of PTCHD4 in WT at passage 2 (P2), negative control transfection at passage 2 (P2-NC) and PTCHD4 OE MEFs at passage 2 (PTCHD4 OE-P2) (n=3). (B-C) Representative images and quantification of EdU and SA-β-galactosidase staining in WT at passage 2 (P2), WT at passage 8 (P8) and PTCHD4 OE MEFs at passage 5 (PTCHD4 OE-P5) (n=3). Scale bars, 50 μm. (D) RT-qPCR analysis of PTCHD4 mRNA expression in P2, P8 and PTCHD4 OE P5 (n=3). (E-F) RT-qPCR analysis of IL-6 and Cxcl2 mRNA expression in P2, P8 and PTCHD4 OE-P5 (n=3). (G) Representative western blot images and quantification of PTCHD4, p53 and p16^INK4A^ in P2, P8 and PTCHD4 OE-P5 (n=3).

**Figure S**6 **PTCHD4 overexpression promotes** senescence-associated **phenotypes in AEC2 cells.**

(A) Representative western blot images and quantitative analysis of PTCHD4, p53 and p21 in PTCHD4-overexpressing AEC2 cells (n=3). (B) RT-qPCR analysis of PTCHD4 mRNA expression in PTCHD4-overexpressing AEC2 cells (n=3). (C-D) Representative images and quantification of EdU and SA-β-galactosidase staining in PTCHD4-overexpressing AEC2 cells (n=3). Scale bars, 50 μm. (E-F) RT-qPCR analysis of IL-6 and Cxcl2 mRNA expression in PTCHD4-overexpressing AEC2 cells (n=3).

Figure S7 Grip strength assessment in young and old WT and PTCHD4^-/-^ mice

Representative grip strength traces of WT and PTCHD4^-/-^ mice in young and old groups (n=6). Each peak represents a single forelimb grip trial, and peak force values were recorded as grip strength. Young and old mice were subjected to identical testing procedures.

Figure S8 Age-associated histological changes in multiple organs of WT and PTCHD4^-/-^ mice.

Representative HE-stained sections of lung, liver, kidney, heart, spleen, skeletal muscle, and brain from young and old WT and PTCHD 4^-/-^ mice (n=6). Scale bars, 50 μm.

Figure S9 Fibrotic features in multiple organs of young and aged WT and PTCHD4^-/-^ mice

Representative Masson’s trichrome-stained sections of lung, liver, kidney, heart, spleen, skeletal muscle, and brain from young and old WT and PTCHD4^-/-^ mice (n=6). Quantification of fibrotic area is shown on the right. Scale bars, 50 μm.

Figure S10 PTCHD4 deficiency attenuates pulmonary fibrosis.

Representative and quantification of Masson’s trichrome-stained lung sections from WT, WT-IPF and PTCHD4^-/-^-IPF mice (n=6) subjected to pulmonary fibrosis. Scale bars, 50 μm.

Figure S11 PTCHD4 deficiency attenuates fibrosis-associated senescence and inflammation in bleomycin-induced pulmonary fibrosis.

(A) Representative immunofluorescence images of lung sections from WT, bleomycin-induced pulmonary fibrosis (IPF), and PTCHD4^-/-^ IPF mice stained for DAPI, CD31, COL1A1 and α-SMA. (B) Representative immunofluorescence images showing co-staining of SFTPC and p21 in lung sections from WT, IPF, and PTCHD4^-/-^ IPF mice. (C-D) Relative mRNA expression levels of IL-6 and Cxcl2 in lung tissues from WT, IPF and PTCHD4^-/-^ IPF mice. (E) Representative immunofluorescence images showing co-staining of α-SMA and p21 in lung sections from WT, IPF and PTCHD4^-/-^ IPF mice. Scale bar, 50 μm.

Figure S12 Inhibition of Akt alleviates the cellular senescence

(A) Representative images and quantification of Akt, p-Akt (S473) in WT-young (WT MEFs at passage2), WT-Old+Ctl (WT MEFs at passage 8), WT-Old+Akt DN (WT MEFs transfected with Akt DN plasma at passage 8) (n=3). (B-C) Representative images and quantification of SA-β-gal and EdU staining in Young (WT MEFs at passage 2), Old (WT MEFs at passage 8), Old+Akt DN (WT MEFs transfected with Akt DN plasma at passage 8), Old+MK2206 (WT MEFs treated with MK2206). (D-E) mRNA expression level of IL-6, Cxcl2 in Young, Old, Old+Akt DN, Old+MK2206 (n=3).

Figure S13 PTCHD4 promotes AKT activation in AEC2 cells.

(A) Representative Western blot images and quantification of p-AKT/Akt levels in AEC2 cells following IR or bleomycin treatment. β-actin was used as the loading control (n=3). (B) Representative Western blot images and quantification of p-AKT/Akt levels in AEC2 cells with or without PTCHD4 overexpression. β-actin was used as the loading control (n=3).

Figure S14 PTCHD4-FLAG pull-down/DIA-MS does not show selective enrichment of AKT family proteins.

PTCHD4-FLAG pull-down followed by DIA-MS analysis was performed in AEC2 and HUVEC cells. Data are shown as log_2_ fold enrichment of PTCHD4-FLAG over control.

Table S1 Primary antibody used were listed as follow.

| **Antibody** | **Supplier** | **Catalog No.** | **Dilution** |
| --- | --- | --- | --- |
| Anti-p53 antibody [PAb 240] | abcam | ab26 | 1:200 |
| Anti-p21 antibody [EPR362] | abcam | ab109520 | 1:1000 |
| Anti-CDKN2A/p16INK4a[EPR20418] | abcam | ab211542 | 1:50 |
| PTCHD4 Antibody | NOVUS | 84223 | 1:1000 |
| beta-Actin Antibody | Cell Signaling Technology (CST) | #4967 | 1:1000 |
| Phospho-Akt (Ser473) Antibody | Cell Signaling Technology (CST) | #9271 | 1:2000 |
| Akt Antibody | Cell Signaling Technology (CST) | #9272 | 1:2000 |
| Phospho-FoxO3a (Ser253) Antibody | Cell Signaling Technology (CST) | #9466 | 1:1000 |
| FoxO3a (D19A7) Rabbit Monoclonal Antibody | Cell Signaling Technology (CST) | #12829 | 1:1000 |
| Anti-rabbit IgG, HRP-linked Antibody | Cell Signaling Technology (CST) | #7074 | 1:3000 |
| Anti-mouse IgG, HRP-linked Antibody | Cell Signaling Technology (CST) | #7076 | 1:3000 |

Table S2 The primers used were listed as follow.

| **Species** | **Gene** | **Forward (5'-3')** | **Reverse (5'-3')** |
| --- | --- | --- | --- |
| *Mus musculus* | GAPDH | TGGCCTTCCGTGTTCCTAC | GAGTTGCTGTTGAAGTCGCA |
|  | Gli1 | CACGCATCCCGAGCACC | GTTCCCTCTACCACGCAGAC |
|  | CCND1 | CAACTTCCTCTCCTGCTACCG | CCTTGTTTAGCCAGAGGCCG |
|  | PTCH1 | CGGGGTGTGGTATGGTATGAG | CAGAGGTCAAACACTGGTACA |
|  | PTCHD4 | TCTGCATTTCCATCGCCACT | TCTTTGGTTCTCCGCCATCC |
|  | IL-6 | CTGCAAGAGACTTCCATCCAG | AGTGGTATAGACAGGTCTGTTGG |
|  | Cxcl2 | CATCCAGAGCTTGAGTGTGACG | GGCTTCAGGGTCAAGGCAAACT |
| *Homo sapiens* | PTCHD4 | TAGTAGGAGCGCATGGAGGT | CGGCCGTCTTAAAAAGCACA |
|  | IL-6 | ACTCACCTCTTCAGAACGAATTG | CCATCTTTGGAAGGTTCAGGTTG |
|  | IL-8 | TTTTGCCAAGGAGTGCTAAAGA | AACCCTCTGCACCCAGTTTTC |

Table S3 Genotyping primers for PTCHD4^-/-^ mice

| **Target allele** | **Primer** | **Sequence (5'->3')** | **Expected product (bp)** |
| --- | --- | --- | --- |
| Mutant allele | P1 | CTGCTGGAGAAGGAGACTGGAGA | 673 |
| Mutant allele | P2 | TGTCTGGCTGGGAGAAAACTGATA |  |
| WT allele  (internal control) | P3 | GGCCAAGATCCCTAGAGAGG | 294 |
| WT allele (internal control) | P4 | GGAGACCCTGCATGACGAAC |  |
